# CircRNA circ-ATAD1 suppresses miR-618 maturation to participate in colorectal cancer

**DOI:** 10.1186/s12876-022-02183-3

**Published:** 2022-05-03

**Authors:** Li Cao, Guanglong Dong, Huan Li

**Affiliations:** 1 Department of Hepatology Surgery, Department of Liver Medicine, Fifth Medical Center, General Hospital of PLA, Beijing, 100039 People’s Republic of China; 2 Department of General Surgery, The General Hospital of People’s Liberation Army, No. 28 Fuxing Road, Haidian District, Beijing, 100853 People’s Republic of China; 3 Military Science Press, Beijing, 100091 People’s Republic of China

**Keywords:** Colorectal cancer, Circ-ATAD1, miR-618, Maturation

## Abstract

**Background:**

CircRNA circ-ATAD1 has been characterized as an oncogenic circRNA in gastric cancer, while its role in colorectal cancer (CRC) is unknown. This study was carried out to explore the role of circ-ATAD1 in CRC.

**Methods:**

Paired CRC and adjacent non-tumor tissue samples collected from 64 CRC patients were subjected to RNA extractions and RT-qPCRs to analyze the expression of circ-ATAD1, premature miR-618, and mature miR-618 in CRC. The effects of circ-ATAD1 overexpression on miR-618 maturation were analyzed by transfecting circ-ATAD1 expression vector into CRC cells, followed by determining the expression of premature miR-618 and mature miR-618 using RT-qPCR. The subcellular location of circ-ATAD1 was analyzed by nuclear fractionation assay, and the interaction between circ-ATAD1 and premature miR-618 was analyzed by RNA pull-down assay. The roles of circ-ATAD1, premature miR-618, and mature miR-618 in regulating CRC cell proliferation were explored by CCK-8 assay.

**Results:**

Circ-ATAD1 was upregulated in CRC and predicted poor survival. In addition, circ-ATAD1 was inversely correlated with mature miR-618 but not premature miR-618. In CRC cells, circ-ATAD1 overexpression decreased the level of mature miR-618 but not premature miR-618. Circ-ATAD1 was detected in both the nucleus and cytoplasm. A direct interaction between circ-ATAD1 and miR-618 was observed. Moreover, circ-ATAD1 overexpression reduced the inhibitory effects of miR-618 overexpression on cell proliferation.

**Conclusion:**

Circ-ATAD1 is overexpressed in CRC and may suppress miR-618 maturation to participate in CRC.

## Background

Colorectal cancer (CRC), also known as rectal cancer, colon cancer, or bowel cancer, is one of the most common types of malignancy in clinical practice [1, 2]. It is estimated that one out of 21 males and one out of 23 females will develop CRC during their lifetime [3]. In recent years, decreased incidence of CRC among patients older than 50 years has been observed [1, 2]. However, its incidence in the young population showed an increasing trend [1, 2]. Despite the advances in the treatment and prevention of CRC, CRC is still a major cause of cancer deaths worldwide [4], mainly due to the lack of cures for metastatic CRC and the low early diagnostic rate [5, 6]. Therefore, novel therapeutic and diagnostic approaches are needed.

Smoking, alcohol abuse, poor dietary structure, and obesity are the major risk factors for CRC [7]. Many molecular players have been known to participate CRC development and progression [8] and have therapeutic potentials in the treatment of CRC as targets of molecular target therapy to regulate gene expression [9, 10]. However, molecular target therapy is still under research and effective targets remain lacking [11]. Despite the lack of protein-coding capacity, circular RNAs (circRNAs, closed by covalent bonds) play critical roles in cancer biology mainly by regulating related gene expression [12, 13]. Therefore, circRNAs are potential targets for molecular target therapy. However, the function of most circRNAs in CRC remains unclear. CircRNA circ-ATAD1 has been characterized as an oncogenic circRNA in gastric cancer [14], while its role in CRC is unknown. Our preliminary microarray analysis has revealed an altered circ-ATAD1 expression in CRC as well as an inverse correlation between circ-ATAD1 and miR-618 (data not shown), a crucial player in cancer biology [15]. In addition, our bioinformatics analysis also revealed the potential interaction between circ-ATAD1 and premature miR-618 (shown in results section). Therefore, we carried out the study to explore the interactions between circ-ATAD1 and miR-618 in CRC.

## Materials and methods

### Patients and follow-up

Ethics Committee of The General Hospital of People’s Liberation Army approved this study before the admission of patients. 64 CRC patients (38 males and 26 females, at the age of 47–67 years, with a mean of 55.1 ± 6.7 years) were enrolled from June 2013 to June 2015. CRC was diagnosed by histopathological biopsy. Patients with initiated therapy, recurrent CRC, and other severe clinical disorders were excluded. Based on the AJCC system, the 64 CRC patients were grouped into stage I (n = 12), II (n = 16), III (n = 20), and IV (n = 16). The 64 patients were followed up monthly for 5 years from the day of admission to record their survival status. Patients who died of causes unrelated to CRC were excluded from the 64 CRC patients included in this study. Informed consent was provided by all patients.

### CRC biopsies and cell lines

Paired CRC and adjacent (within 3 cm around tumors) non-tumor tissues were collected from the 64 patients using the fine needle aspiration method. Following histopathological confirmation, tissue samples were stored in liquid nitrogen prior to the subsequent assays.

WiDr and HT-29 CRC cell lines (ATCC, USA) were used as cell CRC models and cultured in a medium composed of 10%FBS and 90% McCoy's 5a Medium at 37 °C, 95% humidity, and 5% CO_2_. Cells at about 85% confluence were collected and used in the subsequent assays.

Overexpression of circ-ATAD1 and miR-618 in WiDr and HT-29 cells were achieved by transfecting circ-ATAD1 expression vector (1 μg) or miR-618 (50 nM) mimic into 10^8^ WiDr or HT-29 cells through transient transfections mediated by Lipofectamine 2000 (Invitrogen). The expression vector was constructed by Sangon (Shanghai, China) using pcDNA3.1 vector (Invitrogen) as the backbone. MiR-618 mimic and negative control (NC) miRNA were provided by Sigma-Aldrich. Control (C, untransfected cells) and NC (cells transfected with empty vector or NC miRNA) cells were included in each experiment. Transfected cells were cultured in a fresh medium for 48 h prior to the subsequent assays.

### RNA sample preparation

Total RNA was extracted from tissue samples and in vitro cultured cells using RNAzol (Sigma-Aldrich) and treated with DNase I (Invitrogen) to digest genomic DNA for 100 min at 37 °C. RNA integrity and purity were analyzed by electrophoresis on 5% urea-PAGE gels and OD 260/280 ratio, respectively.

### RT-qPCRs

RNA samples with an OD260/280 ratio close to 2.0 (pure RNA) and satisfactory integrity were reverse transcribed (RTs) into cDNA samples using SS-IV-RT system (Invitrogen). With 18S rRNA as an endogenous control, qPCRs were performed using SYBR Green Master Mix (Bio-Rad) to determine circ-ATAD1 expression. Expression of premature miR-618 and mature miR-618 was analyzed using All-in-One™ miRNA qRT-PCR reagent kit (GeneCopoeia). RT-qPCR was performed using poly (T) for premature miR-618 after addition of poly (A) and using sequence-specific primers for mature miR-618 at conditions of 95 °C for 1 min followed by 40 cycles of 95 °C for 10 s and 58.5 °C for 50 s. The primers were 5′-CAGCTGCCAGGTGTTCTT-3′ and 5′-ACCACAGCCT GGAGGCCCA-3′ for circ-ATAD1; 5′-CTACCACATCCAAGGAAGCA-3′ and 5′-TTTT TCGTCACTACCTCCCCG-3′ for human 18S rRNA; 5′-CTCTTGTTCACAGCCAAAC T-3′ and 5′-TCAGACTCATCCACAGGGTA-3′ (reverse) for premature miR-618, 5′-AAACTCTACTTGTCCTTCTGT-3′ and universal reverse primer for miR-618, and 5′-CTCGCTTCGGCAGCACA-3′ and 5′-AACGCTTCACGAATTTGCGT-3′ for U6. Ct values of target genes were normalized to endogenous controls using the 2^−ΔΔCt^ method.

### Nuclear fractionation assay

Nuclear and cytosol fractions were prepared with a Nuclear/Cytosol Fractionation Kit (BioVision, #K266) following the manufacturer’s instructions. After that, RNA isolations and RT-PCR were performed to determine the expression of circ-ATAD1 in both fractions.

### RNA pull-down assay

Premature miR-618 and NC miRNA were ligated to biotin (Bio-miR-618 (pre) and Bio-NC) and transfected into WiDr and HT-29 cells. After 48 h of transfection, miR-618 was pull down by streptavidin agarose magnetic beads (Life Technologie). After that, RNA isolations and RT-qPCRs were performed to determine circ-ATAD1 expression.

### Cell counting kit-8 (CCK-8) assay

WiDr and HT-29 cells with transfections were subjected to cell proliferation analysis using a CCK-8 kit from Dojindo (Japan). In brief, cells were counted and transferred to a 96-well cell culture plate with 3000 cells in 0.1 ml fresh medium per well. Three replicate wells were set for each experiment. Cells were cultured as mentioned above, followed by determining OD values (450 nm) every 24 h until 96 h. At 2 h prior to the determination of OD values, CCK-8 solution was added to reach the final concentration of 10%.

### Colony formation assay

Transfected cells were seeded to six-well plates and cultured at 37 °C and 5% CO_2_ incubator for 2 weeks. After that, cells were fixed using 10% formalin (Sigma), stained with Giemsa (Sigma), and photographed.

### Statistical analysis

Paired t test was applied to analyze the differential expression of genes in CRC and non-tumor tissues. Comparisons among multiple independent cell transfection groups were analyzed by ANOVA Tukey’s test. Correlations between the expression of genes were analyzed by Pearson’ correlation coefficient. To analyze the prognostic value of circ-ATAD1 for CRC, the 64 CRC patients were grouped into high and low circ-ATAD1 level groups (n = 32, cutoff value = the median level of circ-ATAD1 expression in CRC tissues). Survival curves were plotted based on the 5-year follow-up data and compared by log-rank test. P < 0.05 was deemed statistically significant.

## Results

### Circ-ATAD1 was overexpressed in CRC and predicted poor survival

**Fig. 1:**
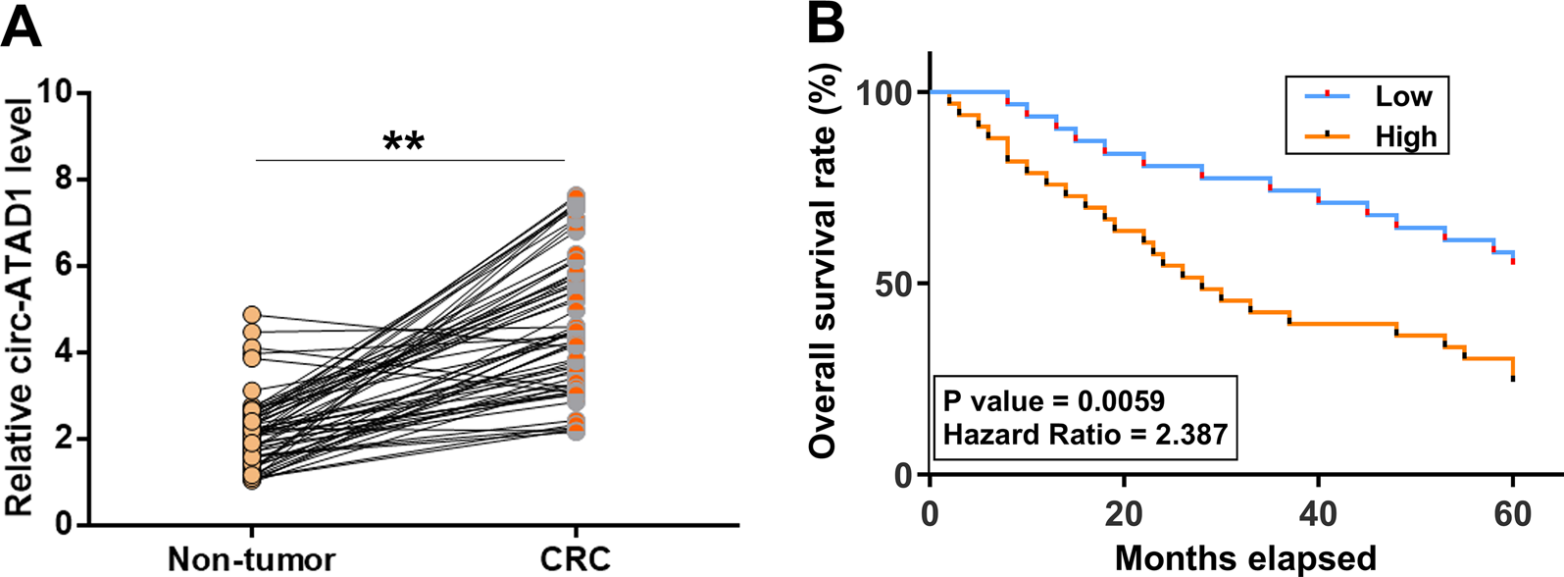
Circ-ATAD1 was overexpressed in CRC and predicted poor survival. Paired CRC and non-tumor tissues collected from CRC patients (n = 64) were subjected to RNA extractions and RT-qPCRs to determine the expression of circ-ATAD1. Average values of three qPCR technical replicates were compared (**a**). To analyze the prognostic value of circ-ATAD1 for CRC, the 64 CRC patients were grouped into high and low circ-ATAD1 level groups (n = 32, cutoff value = the median level of circ-ATAD1 expression in CRC tissues). Survival curves were plotted based on the 5-year follow-up data and compared by log-rank test (**b**). **p < 0.01

Paired CRC and non-tumor tissues collected from CRC patients (n = 64) were subjected to RNA extractions and RT-qPCRs to determine circ-ATAD1 expression. Paired t test analysis showed that circ-ATAD1 expression was upregulated in CRC tissues compared to non-tumor tissues (Fig. 1a). Survival analysis showed that patients in the high circ-ATAD1 level group experienced a significantly lower overall survival rate compared to patients in the low circ-ATAD1 level group (Fig. 1b, p < 0.01). Chi-squared analysis showed that circ-ATAD1 expression in CRC tissues was closely correlated with patients’ clinical stages and tumor size, but not with patients’ age, gender, tumor location, and distance tumor metastasis (Table 1).

**Table 1:** Chi-squared analysis of the associations between patients’ clinical data and circ-ATAD1 expression in CRC tissues

Parameter	Total	circ-ATAD1		Chi square	P
		Low (n = 32)	High (n = 32)		
*Age (years)*					
< 55	33	15	18	0.56	0.45
> = 55	31	17	14		
*Gender*					
Female	38	20	18	0.26	0.21
Male	26	12	14		
*Location*					
Rectum	36	20	16	1.02	0.31
Colon	28	12	16		
*Clinical stage*					
I/II	28	18	10	4.06	0.04
III/IV	36	14	22		
*Tumor size*					
< 5 cm	26	19	7	9.33	0.002
> = 5 cm	38	13	25		
*Distant metastasis*					
No	49	27	22	2.18	0.14
Yes	15	5	10		

### CRC tissues exhibited downregulated mature miR-618 but not premature miR-618

**Fig. 2:**
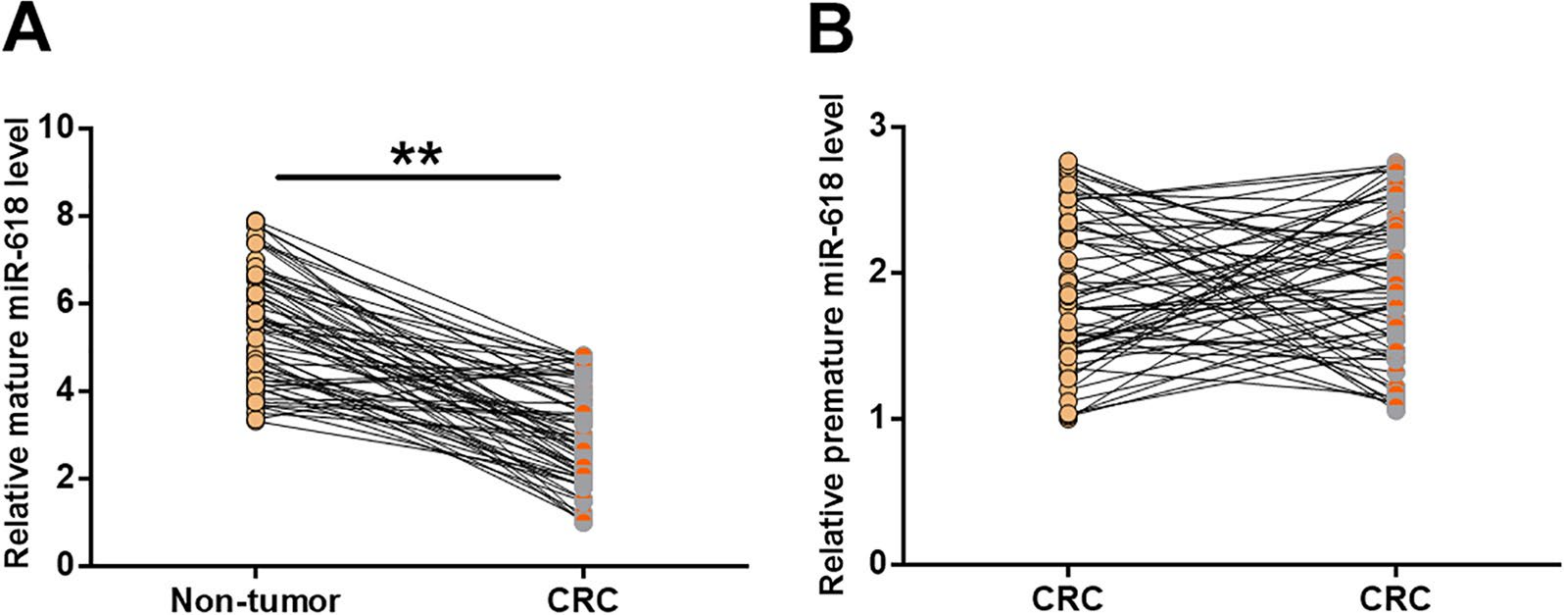
CRC tissues exhibited downregulated mature miR-618, but not premature miR-618. Expression of mature miR-618 (**a**) and premature miR-618 (**b**) in the paired tissue samples from 64 CRC patients was also analyzed by RT-qPCR. Differential expression of mature miR-618 and premature miR-618 was analyzed by paired t test. **p < 0.01

Expressions of mature miR-618 and premature miR-618 in the paired tissue samples from 64 CRC patients were also analyzed by RT-qPCR. Paired t test analysis showed that miR-618 expression was upregulated in CRC tissues compared to non-tumor tissues (Fig. 2a, p < 0.01). In contrast, premature miR-618 showed no significant difference in CRC and non-tumor tissue samples (Fig. 2b). Therefore, miR-618 maturation may participate in CRC.

### Circ-ATAD1 was inversely correlated with mature miR-618 across CRC tissue samples

**Fig. 3:**
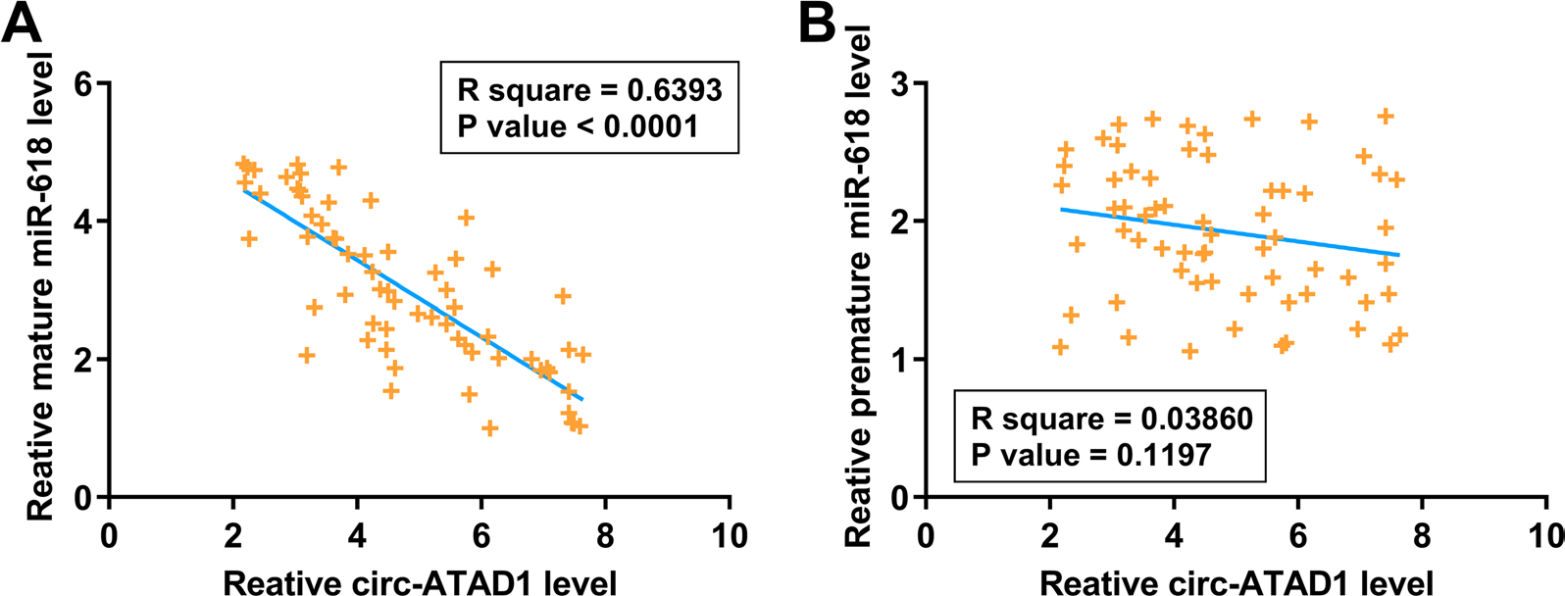
Circ-ATAD1 was inversely correlated with mature miR-618 across CRC tissue samples. Pearson’s correlation coefficient was performed to analyze the correlations between circ-ATAD1 and mature miR-618 or premature miR-618 across CRC tissue samples

Pearson’s correlation coefficient was performed to analyze the correlations between circ-ATAD1 and mature miR-618 or premature miR-618 across CRC tissue samples. It was observed that circ-ATAD1 was significantly and inversely correlated with mature miR-618 across tumor tissues (Fig. 3a). In contrast, no significant correlation between circ-ATAD1 and premature miR-618 was observed across non-tumor tissues (Fig. 3b). Therefore, circ-ATAD1 is likely related to miR-618 maturation.

### Circ-ATAD1 overexpression suppressed miR-618 maturation in WiDr and HT-29 cells

**Fig. 4:**
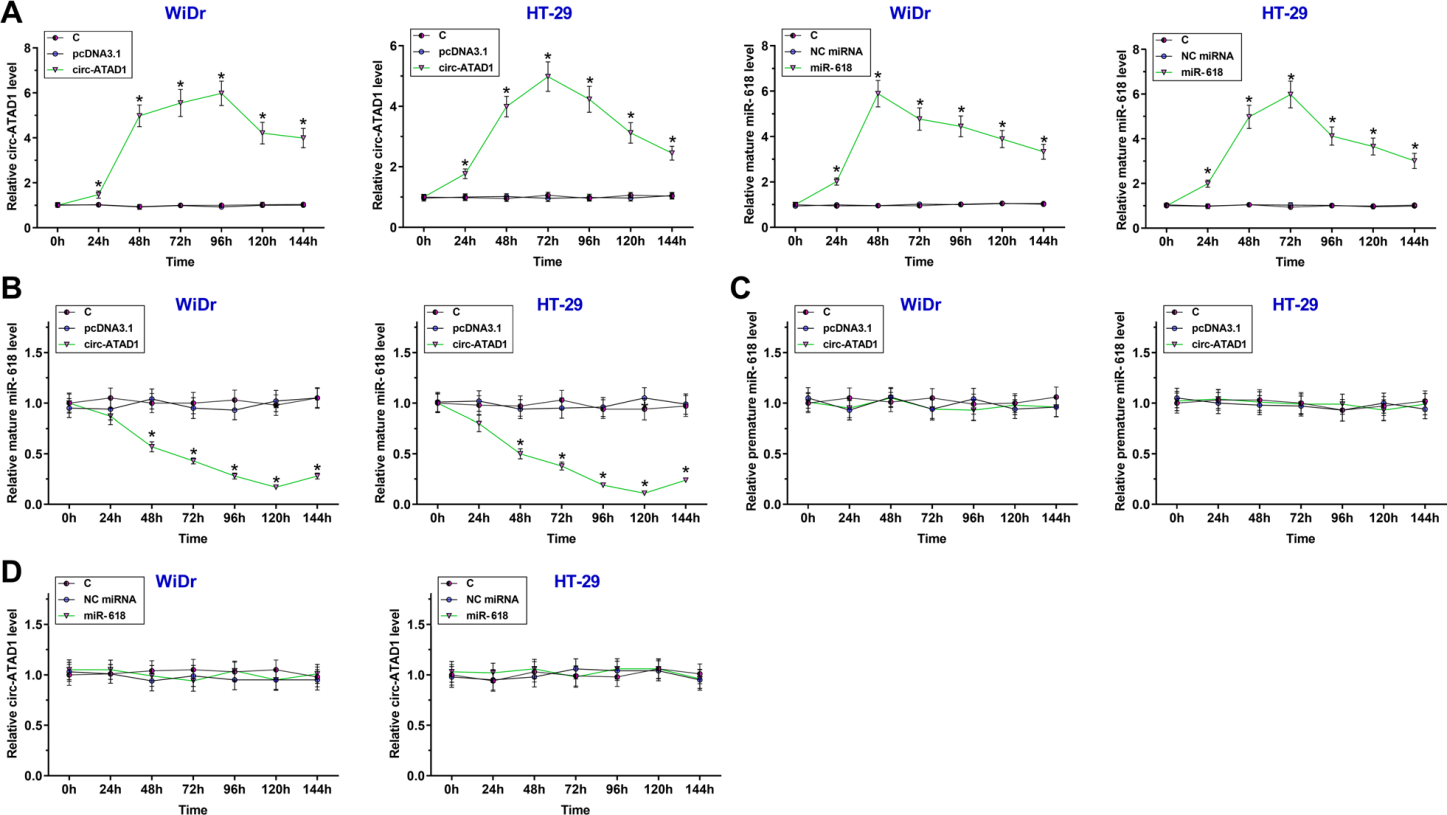
Circ-ATAD1 overexpression suppressed miR-618 maturation in WiDr and HT-29 cells. To analyze the effects of circ-ATAD1 on miR-618 maturation, WiDr and HT-29 cells were transfected with either circ-ATAD1 expression vector or miR-618 mimic, followed by determining the expression of circ-ATAD1 and miR-618 every 24 h until 144 h using tRT-qPCRs (**a**). The effects of circ-ATAD1 overexpression on the expression of mature miR-618 (**b**) and premature miR-618 (**c**), and the effects of mature miR-618 overexpression on circ-ATAD1 expression (**d**) were analyzed by RT-qPCRs. *p < 0.05

To analyze the effects of circ-ATAD1 on miR-618 maturation, WiDr and HT-29 cells were transfected with either circ-ATAD1 expression vector or miR-618 mimic, followed by determining the expression of circ-ATAD1 and miR-618 every 24 h until 144 h using RT-qPCRs. It was observed that circ-ATAD1 and mature miR-618 were significantly overexpressed between 24 and 144 h (Fig. 4a, p < 0.05). Interestingly, circ-ATAD1 overexpression decreased mature miR-618 expression (Fig. 4b, p < 0.05), but not premature miR-618 (Fig. 4c). Moreover, miR-618 overexpression failed to significantly affect the expression of circ-ATAD1 (Fig. 4d).

### Circ-ATAD1 increased the proliferation of WiDr and HT-29 cells through miR-618

**Fig. 5:**
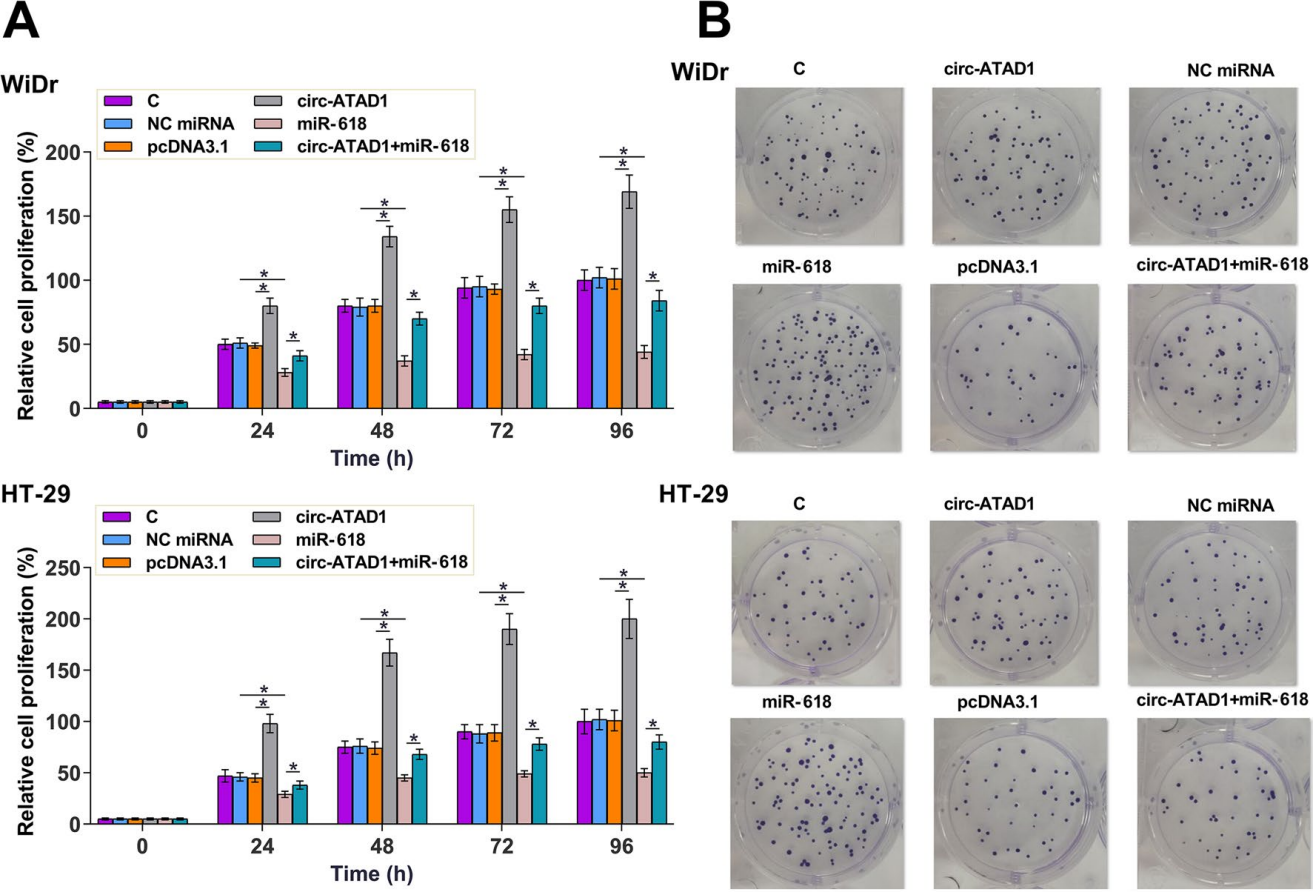
Circ-ATAD1 expression increased the proliferation of WiDr and HT-29 cells via miR-618. The role of circ-ATAD1 and miR-618 in regulating the proliferation of WiDr and HT-29 cells was analyzed by CCK-8 assay. Cell proliferation was monitored by measuring OD values at 450 nm every 24 h until 96 h (**a**). The results of CCK-8 were further verified by colony formation assay (**b**). *p < 0.05

The role of circ-ATAD1 and miR-618 in regulating the proliferation of WiDr and HT-29 cells was analyzed by CCK-8 assay. It was observed that circ-ATAD1 overexpression increased cell proliferation and miR-618 overexpression decreased cell proliferation. Moreover, circ-ATAD1 overexpression reduced the inhibitory effects of miR-618 overexpression on cell proliferation (Fig. 5a, p < 0.05). A colony formation assay was applied to verify the results of CCK-8 assay. Similar results were obtained (Fig. 5b).

### Circ-ATAD1 is localized in both nucleus and cytoplasm and directly interacts with premature miR-618

**Fig. 6:**
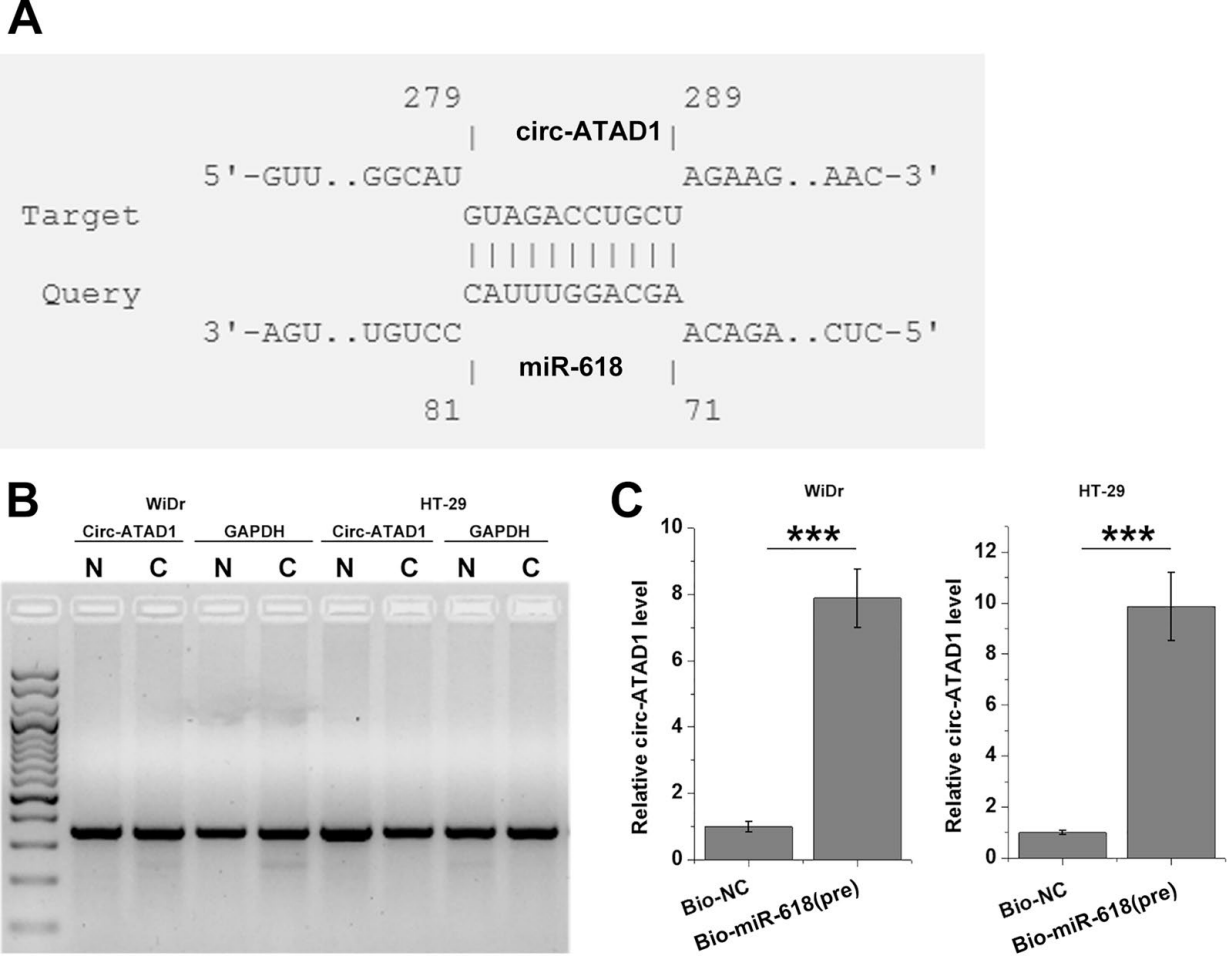
Circ-ATAD1 is localized to both nucleus and cytoplasm and directly interacts with premature miR-618. The potential base-pairing formed by circ-ATAD1 and premature miR-618 was predicted by IntaRNA 2.0 (**a**). Nuclear fractionation assay was used to determine circ-ATAD1 expression in both nuclear (N) and cytosol (C) fractions of WiDr and HT-29 cells (**a**). RNA pull-down assay was performed to analyze the direct interaction between circ-ATAD1 and premature miR-618 (**b**). ***p < 0.001

The potential base-pairing formed by circ-ATAD1 and premature miR-618 was predicted by IntaRNA 2.0 [16]. It was predicted that circ-ATAD1 and miR-618 could form multiple base pairs (Fig. 6a). Nuclear fractionation assay was used to determine the expression of circ-ATAD1 in both nuclear (N) and cytosol (C) fractions of WiDr and HT-29 cells. It was observed that circ-ATAD1 was expressed in both fractions (Fig. 6b). RNA pull-down assay was performed to analyze the direct interaction between circ-ATAD1 and premature miR-618. Compared to Bio-NC group, Bio-miR-618 (pre) group showed significantly increased circ-ATAD1 expression (Fig. 6c, p < 0.001), suggesting the direct interaction between them.

## Discussion

This study mainly investigated the involvement of circ-ATAD1 and miR-618 in CRC and explored their interaction. The results showed that circ-ATAD1 was significantly upregulated in CRC and might downregulate the expression of mature miR-618 to promote CRC cell proliferation.

The role of circ-ATAD1 in cancer biology has only been explored in gastric cancer [14]. It has been reported that circ-ATAD1 is overexpressed in gastric cancer and sponges miR-140-3p to upregulate YY1, thereby increasing cancer cell proliferation [14]. Based on our knowledge, the role of circ-ATAD1 in other cancers is unknown. In this study, we first reported the upregulation of circ-ATAD1 in CRC and its enhancing effects on CRC cell proliferation. Therefore, circ-ATAD1 is likely an oncogenic lncRNA in CRC.

Treatment of CRC in clinical practice is challenged by the poor prognosis of metastatic cases [5, 6]. Unfortunately, early diagnosis of CRC is unlikely to be significantly improved in the near future, mainly owing to the lack of sensitive diagnostic biomarkers. In this study, we showed that high circ-ATAD1 levels were correlated to the poor survival of CRC patients. Therefore, monitoring circ-ATAD1 expression in CRC patients may guide the determination of therapeutic approaches, which in turn improve patients’ survival. However, the prognostic value of circ-ATAD1 remains to be further analyzed.

MiR-618 has been characterized as a tumor suppressor in different cancers [15, 17]. For instance, miR-618 is downregulated in prostate cancer and suppresses cancer cell invasion and migration by targeting FOXP2 [15]. Although the function of miR-618 in CRC is unknown, it is known that the susceptibility of CRC in a Han Chinese population is correlated with the polymorphism rs2682818 in miR-618 [15]. In this study, we showed that the decreased maturation of miR-618, but not the transcription of miR-618 gene, is involved in CRC. In addition, circ-ATAD1 overexpression is likely responsible for the reduced production of mature miR-618 in CRC cells. We speculated that circ-ATAD1 could suppress the transportation of premature miR-618 from the nucleus to the cytoplasm, which is required for miR-618 maturation. To form mature miRNAs, premature miRNAs are first transported from the nucleus to the cytoplasm [18, 19]. We showed that circ-ATAD1 was detected in both nucleus and cytoplasm and directly interacted with premature miR-618. Therefore, our data supported the hypothesis that circ-ATAD1 could sponge premature miR-618 in the nucleus to suppress its transportation, thereby reducing miR-618 maturation.

## Conclusion

Circ-ATAD1 is upregulated in CRC and may suppress miR-618 maturation to promote CRC cell proliferation.

## Data Availability

The data was available from corresponding author upon reasonable request.

## Abbreviation

CRC: Colorectal cancer
